# Employing a mobile health decision aid to improve decision-making for patients with advanced prostate cancer and their decision partners/proxies: the CHAMPION randomized controlled trial study design

**DOI:** 10.1186/s13063-021-05602-0

**Published:** 2021-09-16

**Authors:** Lourdes R. Carhuapoma, Winter M. Thayer, Catherine E. Elmore, Jane Gildersleeve, Tanmay Singh, Farah Shaukat, Melissa K. Uveges, Tamryn Gray, Crystal Chu, Daniel Song, Patricia J. Hollen, Jennifer Wenzel, Randy A. Jones

**Affiliations:** 1 University of Virginia School of Nursing, 202 Jeanette Lancaster Way, PO Box 800782, Charlottesville, VA 22908 USA; 2 Division of Neurosciences Critical Care, Johns Hopkins Hospital, 1800 Orleans Street, Baltimore, MD 21287 USA; 3 Johns Hopkins University School of Nursing, 525 N. Wolfe Street, Baltimore, Maryland 21205 USA; 4 University of Virginia School of Nursing, 202 Jeanette Lancaster Way, PO Box 800782, Charlottesville, VA 22908 USA; 5 University of Virginia School of Nursing, 202 Jeanette Lancaster Way, PO Box 800782, Charlottesville, VA 22908 USA; 6 Department of Radiation Oncology, Johns Hopkins University School of Medicine, 401 North Broadway, Baltimore, MD 21231 USA; 7 Johns Hopkins University School of Nursing, 525 N. Wolfe Street, Baltimore, Maryland 21205 USA; 8 Boston College Connell School of Nursing, Maloney Hall 375, 140 Commonwealth Avenue, Chestnut Hill, MA USA; 9 Palliative Care, Dana Farber Cancer Institute, 375 Longwood Avenue, Boston, MA 02215 USA; 10 University of Virginia School of Nursing, 202 Jeanette Lancaster Way, PO Box 800782, Charlottesville, VA 22908 USA; 11 Department of Radiation Oncology, Johns Hopkins University School of Medicine, 401 North Broadway, Baltimore, MD 21231 USA; 12 University of Virginia School of Nursing, 202 Jeanette Lancaster Way, PO Box 800782, Charlottesville, VA 22908 USA; 13 Johns Hopkins University School of Nursing, 525 N. Wolfe Street, Baltimore, Maryland 21205 USA; 14 University of Virginia School of Nursing, 202 Jeanette Lancaster Way, PO Box 800782, Charlottesville, VA 22908 USA

**Keywords:** Decision-making, Advanced prostate cancer, Decision aid, Minorities, mHealth, Community patient navigator

## Abstract

**Background:**

Metastatic prostate cancer remains a lethal malignancy that warrants novel supportive interventions for patients and their decision partners and proxies. Decision aids have been applied primarily to patients with localized disease, with minimal inclusion of patients with advanced prostate cancer and their decision partners. The use of a community patient navigator (CPN) has been shown to have a positive supportive role in health care, particularly with individuals from minority populations. Research is needed to evaluate decision support interventions tailored to the needs of advanced prostate cancer patients and their decision partners in diverse populations.

**Methods:**

Guided by Janis and Mann’s Conflict Model of Decision Making, the Cancer Health Aid to Manage Preferences and Improve Outcomes through Navigation (CHAMPION) is a randomized controlled trial to assess the feasibility and acceptability of a mobile health (mHealth), CPN-administered decision support intervention designed to facilitate communication between patients, their decision partners, and the healthcare team. Adult prostate cancer patients and their decision partners at three mid-Atlantic hospitals in the USA were randomized to receive enhanced usual care or the decision intervention. The CHAMPION intervention includes a theory-based decision-making process tutorial, immediate and health-related quality of life graphical summaries over time (using mHealth), values clarification via a balance sheet procedure with the CPN support during difficult decisions, and facilitated discussions with providers to enhance informed, shared decision-making.

**Discussion:**

The CHAMPION intervention is designed to leverage dynamic resources, such as CPN teams, mHealth technology, and theory-based information, to support decision-making for advanced prostate cancer patients and their decision partners. This intervention is intended to engage decision partners in addition to patients and represents a novel, sustainable, and scalable way to build on individual and community strengths. Patients from minority populations, in particular, may face unique challenges during clinical communication. CHAMPION emphasizes the inclusion of decision partners and CPNs as facilitators to help address these barriers to care. Thus, the CHAMPION intervention has the potential to positively impact patient and decision partner well-being by reducing decisional conflict and decision regret related to complex, treatment-based decisions, and to reduce cancer health disparities.

Trial registration

ClinicalTrials.govNCT03327103. Registered on 31 October 2017—retrospectively registered. World Health Organization Trial Registration Data Set included in Supplementary Materials.

**Supplementary Information:**

The online version contains supplementary material available at 10.1186/s13063-021-05602-0.

## Background

Recent advances in cancer therapy have contributed to limited knowledge about effective treatment and supportive care interventions for patients with advanced cancer and their decision partners. While a decline in the overall incidence of prostate cancer has been noted in recent years, advanced prostate cancer rates are on the rise. Rates are projected to increase through 2025, primarily among men younger than 70 years of age [1–3]. The annual burden of metastatic prostate cancer in the USA is estimated to increase 42% by 2025 [3]. Recent advances in cancer therapy have led to increased survival for many prostate cancer patients [4]. However, this benefit expands the need for knowledge about effective supportive care interventions for patients with advanced cancer and their decision partners. Despite improvements in cancer therapy, advanced prostate cancer remains a life-limiting disease that warrants novel supportive care interventions, particularly for patients with high disease burden facing complex, treatment-based decisions during the cancer process.

Treatment decisions—notably deciding when to start, change, or stop treatment—can overwhelm patients and their decisions partners and proxies. Decision partners have been identified as an important source of support for patients at these crucial decision points [5, 6]. A decision partner is a person who has a relationship with the patient and maintains a willingness to participate in decision making or proxy decision-making, understands the patient’s condition and anticipated treatment decisions, and demonstrates self-efficacy, the emotional capacity required for participation, as well as a willingness to fulfill several supportive roles, such as patient advocate [7].

When a patient’s disease progresses to an advanced stage and the decision-making capacity wanes, decision partners often step into the role of proxy decision-maker for treatment-related decision-making for their loved ones [8]. Decision partners are ideal proxy decision-makers due to their proximity to the patient and knowledge of the patient’s values and preferences. The proxy decision-making process has been shown to be associated with negative outcomes for the decision partner, including stress, guilt, or depression [9, 10]. Decision partners may experience an immense amount of decision-making burden related to decision-making, particularly when patients have limited decision-making capacity for reasons such as brain metastases or cognitive deficits related to treatment. Studies [11–13] have noted the value of decision partners in helping to make decisions for terminally ill patients when they are most vulnerable [14].

Interventions to increase the engagement of decision partners and enhance shared decision-making in the prostate cancer treatment decision-making process have the potential to (1) reduce decisional conflict and decision regret [15–18] and (2) improve the health-related quality of life (HRQOL) outcomes for patients and their decision partners, particularly in the realm of psychosocial wellness. Decisional conflict is the inclination to accept or reject a course of action at the same time [15]; decision regret is an undesirable, cognitively influenced emotion that is experienced when one wishes that the current situation would have been better if one had made a different decision [18–20]. A decision aid is one type of evidence-based intervention that promotes informed and shared health-related decision-making [21]. Although there is a growing body of literature describing the utility of decision aids in prostate cancer [22–24], to our knowledge, decision aids have been applied primarily to patients with localized disease, with an emphasis on treatment knowledge or options, with minimal inclusion of patients with advanced prostate cancer and their decision partners. Many decision aids are patient-directed; however, this orientation may not be appropriate in advanced cancer states, especially for minority patients who are less likely to make decisions alone [21]. Individuals with advanced cancer from minority groups with advanced cancer, particularly African Americans, are often faced with ineffective communication between themselves and their oncologist. This can lead to a breakdown in the effectiveness of cancer treatments and result in significant distress for the patients and their decision partners [25–27]. Targeted decision support interventions are needed that incorporate commonly noted African American values, such as spirituality, family and trusted others [25–27].

The role of the community patient navigator (CPN) has proliferated due to its demonstrated effectiveness in community outreach, social support, informal counseling, and health education [28]. CPNs have been associated with improved care access and reduced healthcare costs, particularly among minorities [29–31]. Mobile health (mHealth) tools, such as electronic tablets and smartphones, have an important healthcare role, such as the capacity to enhance communication between the oncologist and the patient, particularly those who are underserved [32–34]. mHealth technology can function as a resource to support patients and decision partners, aid in decision making, and help bridge health communication between the patient, decision partner, and oncologist [32, 35, 36]. Despite the growing role of CPNs in cancer care [37, 38] and the utility of interactive mHealth technologies, decision aid use by CPNs to improve patient outcomes has been mostly unexplored. Subsequently, there is an urgent need to describe and evaluate CPN-administered mHealth decision support interventions that are tailored to advanced prostate cancer patients and their decision partners and that address the unique needs of racial and ethnic minority populations.

The Cancer Health Aid to Manage Preferences and Improve Outcomes through Navigation (CHAMPION) study intervention involves an interactive mHealth decision aid delivered by CPNs, and includes measures of health-related QoL throughout the course of the patient’s illness trajectory. The intervention has been pilot tested [39, 40] for advanced prostate cancer patients to enhance informed, shared decision making. In present study, the role of the CPN in the decision-making process will also be assessed. CHAMPION is designed to improve decision-making processes, match patients’ and decision partners’ preference to participate in treatment decisions, enhance patient-provider communication, and enrich psychosocial health-related quality of life (HRQOL-PSY) outcomes for patients and their decision partners. The primary aims of this study are to decrease decisional conflict/uncertainty, decrease decision regret, enhance HRQL-PSY, assess participation preferences in decision-making, and examine potential differences by race (African American versus White and Others) related to using the CHAMPION intervention. Because low decision-making involvement is thought to contribute to cancer-related health disparities, this novel intervention has the potential to positively impact the decision-making process for patients and their decision partners with the inclusion of the CPN, clarifying and supporting the treatment decision-making process.

## Decision-making theoretical framework

Janis and Mann’s Conflict Model of Decision Making underpins the CHAMPION intervention [15, 16, 40]. This theory of decision-making proposes steps to promote good decision-making for consequential decisions in which perceived losses exist, no matter the choice. Stress is recognized as negatively affecting decision-making because decision-making requires high-level cognitive processes. With emphasis on the preconditions of risk, hope, and time by oncology providers, more time for clarifying options, values, and preferences (via use of a decision balance sheet), and increased provider support (structured discussions with providers), patients and their decision partners are supported in the decision-making process and may be less likely to feel regret or dwell on decisions out of their control. This theoretical framework has been applied to the CHAMPION study (a) as a brief decision-making process tutorial taught and reviewed with participants using an easy-recall method and (b) to predict outcomes for the patient-decision partner dyads.

## Methods/design

### Study design, setting, and population

The CHAMPION study is an ongoing randomized control trial to assess the effectiveness of a decision intervention (DI) compared to enhanced usual care (EUC) alone. Participants enter the study at one of three single-event decisions: (1) starting, (2) changing, or (3) stopping anti-cancer treatment. For example, a patient being monitored after primary cancer treatment who is diagnosed with disease progression and offered new treatment options would be eligible for this study. Alternatively, a patient who does not respond to, or experiences side effects from, their first choice of treatment and is now considering changing to a new treatment would also be eligible. An example of stopping treatment is a patient who has been on Androgen Deprivation Therapy and is considering stopping treatment. The unit of randomization is the patient-decision partner dyad, with stratification based upon the patient’s race (African American, White, or Other), and by decision event (starting, changing, or stopping anti-cancer treatment). Dyads will be randomly assigned after baseline data collection using a computerized random number generator weighted to have a greater number of DI than EUC dyads. Dyads will be randomized by a study team member, who will have access to sealed envelopes in the event that the computer system is not operational.

**Fig. 1:**
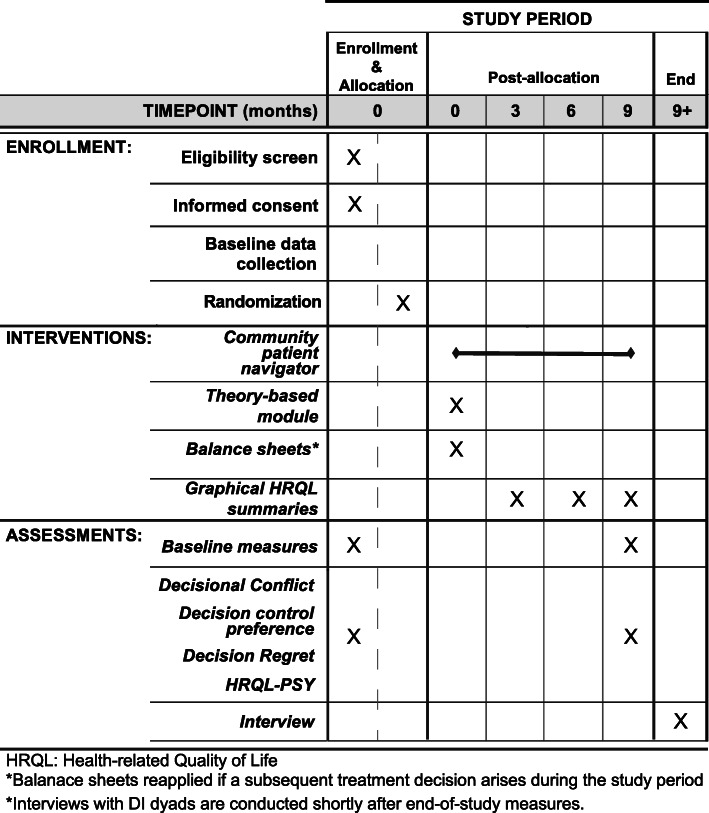
Trial design schematic for schedule of enrollment, interventions, and assessments

**Fig. 2:**
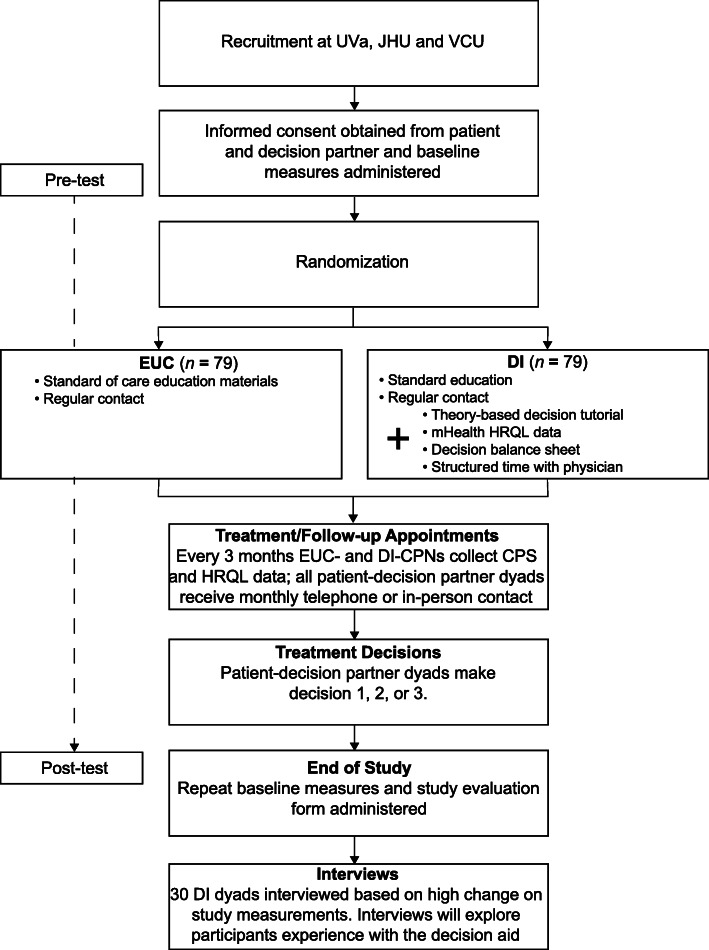
CHAMPION study design flow chart

A pre-test/post-test design (see Fig. 1) for the three single-event decisions (starting, changing, or stopping anti-cancer treatment) is employed to assess change in HRQL-PSY, decisional conflict, and decision regret. Qualitative methods (post-intervention interviews) are combined with quantitative measurement to enhance the understanding of the patient-decision partner dyads’ experiences using the mHealth decision aid, particularly, in regard to their race and decision event. Patients and decision partners are randomly assigned as a dyad to one of two study arms: EUC or DI. EUC participants receive CPN-administered standard-of-care cancer educational materials not specific to decision making. DI participants receive CPN-administered standard-of-care cancer educational materials plus a previously piloted multicomponent intervention that is designed to facilitate decision partner involvement in the decision-making and communication within the patient-decision partner dyad, as well as between the dyad and the oncologist. Figure 2 displays the CHAMPION study design flow chart.

The study setting includes three National Cancer Institute designated academic medical centers in the mid-Atlantic region of the USA: the University of Virginia (UVa), Johns Hopkins University (JHU), and Virginia Commonwealth University (VCU). The UVa Cancer Center predominantly serves rural areas in South, Southwest, Central, and North-Central Virginia [41] with approximately 11% of the catchment area residents being African American and 4% Hispanic. The Sydney Kimmel Comprehensive Cancer Center at JHU draws patients from Baltimore City and the surrounding area, as well as those seeking clinical trials, with approximately 20% of the patients being African American and 3% Hispanic. The VCU Massey Cancer Center predominantly serves rural areas in Central-East and Central-West Virginia [42, 43], with approximately 27% of the catchment area residents being African American and 6% Hispanic.

Patient inclusion criteria include the following: a diagnosis of stage III/IV prostate cancer, life expectancy of ≥ 6 months, Karnofsky Performance Status [44] ≥ 60, age ≥ 18 years, and the ability to understand English. Exclusion criteria include the following: severe psychiatric problem, prior non-prostate malignancy in the past 3 years (except treated basal cell/squamous cell skin cancer or superficial bladder cancer), and cognitive impairment. A s*evere psychiatric problem* is defined as inpatient psychiatric treatment in the past 6 months, current treatment with antipsychotic medication, current hallucinations, and/or current serious thoughts of suicide. Cognitive impairment is defined as an adjusted error score of > 5 on the Short Portable Mental Status Questionnaire or other issues preventing participation. A decision partner is defined in the eligibility criteria as a person that the patient identifies as supporting the patient in the decision-making. Consistent with the literature definitions [7], the decision partner is most often a spouse, but can also be an adult family member, friend, or spiritual advisor. In the present study, participants were asked to identify a single decision partner. For illustrative purposes, a ‘typical’ patient-decision partner dyad in this study appears as described in Table 1.

**Table 1:** A “typical” CHAMPION study dyad (composite case)

	Age	Race	Ethnicity	Insurance	Employment
Patient	67	African American	Non-Hispanic	Public and private	Employed
Decision partner	68	African American	Non-Hispanic	Public	Retired
**Sample case history:** Patient was diagnosed in 2009 with regionally metastatic prostate cancer, underwent surgery to remove the prostate and regional lymph nodes at that time. In 2017, the patient developed an elevated PSA and was diagnosed with bone metastases after imaging and began hormonal treatment. Six months later, the patient experiences bone pain and undergoes additional imaging that shows further metastases. He and his decision partner are approached by the research team to seek consent for participation in CHAMPION following a consultation visit to discuss treatment options with the oncologist. Informed consent is obtained from the patient and the decision partner. Baseline data are collected and the patient-decision partner dyad are enrolled and randomized to the DI arm. The CPN reviews the decision theory and the dyad is introduced to the balance sheet activity via the practice “telling others about cancer” decision. The dyad is sent home with the preloaded mp3 player. Ahead of their return visit, the CPN assesses the dyad’s concerns and conducts the single-event decision (starting, changing or stopping treatment). During the decision visit with the oncologist, there is structured time to discuss any concerns that arose during the balance sheet activity. Over the next 9 months, the CPN maintains regular contact and provides graphical summaries of the patient’s HRQL-PSY symptoms, as reported by both the patient and the decision partner.					

The target sample for this trial is 158 patient-decision partner dyads. Quota sampling will be applied to obtain a study sample of at least 40% African American. Although the intervention was not developed for primary use in the African American population, this target was chosen to facilitate the team’s ability to estimate the impact of the intervention in the African American population. Although prostate cancer has high incidence in African American men, minority participation can be low; thus, recruitment strategies are being applied that focus on African American participants.

### Recruitment

Recruitment began in 2017 at UVa and JHU, and in 2019 at VCU. Participants are screened at each site using a variety of methods, including research team attendance at weekly clinical case discussions, manual chart review, and referrals from the clinical team. In this event-driven design, oncology clinic providers identify relevant events, such as the patient’s need to address starting, changing, or stopping treatment, and then refer eligible patient-decision partner dyads during weekly clinical trials meetings by teleconferences held at all institutions. In addition, the research teams attend weekly case discussions and discuss patients who may meet the eligibility criteria. The research team subsequently reviews these patients’ medical charts to determine eligibility. If a patient is eligible, the research team addresses the opportunity to participate in the study at the patient’s next clinical visit or over the telephone.

After confirming eligibility, the oncologist or another care provider introduces the study to the patient and decision partner. Patients experiencing serologic, radiographic, or symptom progression are contacted by the research team. The majority of patients and/or dyads are approached directly before or after their clinical visit with the medical oncologist. The research team reviews the study and, if the patient and/or the patient-decision partner dyad is willing, engages in an informed consent process to obtain oral consent or a signature from the patient and the decision partner, as approved by the facilities’ institutional review board (IRB). No data is planned to be used for further studies, which would constitute obtaining additional consent from current participants.

A previous version of the protocol required a decision partner for participation, but this requirement was removed after the study began, based on expert advice and research team discussion regarding the additional value of including patients without decision partners in the study. In the decision to include patients without decisional partners, there is a potential limitation in the analyses to compare between those patients who have decisional partners with those without decisional partners. We will plan to examine differences between dyads and single participants, particularly related to decisional conflict, regret, and quality of life.

Once the participant(s) consents to participate, the study team administers the baseline measures and randomizes the patient-decision partner dyad to either the EUC or DI group. Patient and/or patient decision partner dyads are enrolled in the study for approximately nine months. IRB approval is sought and obtained from each of the participating sites.

### Intervention

The CHAMPION intervention incorporates mHealth technology to emphasize informed, shared decision-making for patients, decision partners and healthcare providers through values clarification, involvement preference, and HRQOL feedback [34, 35]. This decision aid is a comprehensive, cognitive-behavioral skills program that utilizes seven key components suggested by the theoretical framework to assist patients and their decision partners with goal-setting, establishing desired decision control, and facilitating discussions between oncology providers. The seven key decision aid components (see Table 2) are applied during any of three single-event decisions during the advanced cancer trajectory: (1) starting, (2) changing, or (3) stopping anti-cancer treatment [40].

**Table 2:** Description of seven key decision aid components [41]

1. **Anticipatory guidance** for managing treatment side effects, quality-of-life and available therapies delivered via pamphlets from National Cancer Institute, Cancer Care National Office, or institutionally distributed.	
2. **Social support** provided by the decision partner’s participation in the study and interactions with the CPN.	
3. **Patient’s preferences for treatment decision-making participation** is assessed and shared with health care providers.	
4. **Decision-making process tutorial** communicates the importance of decision-making and satisfaction via an easy-recall teaching method that addresses (a) the purpose of the decision aid, (b) possible outcomes, (c) preconditions, (d) theory utility, (e) styles of decision making, (f) the steps/process of decision making, and (g) evaluation criteria. This is presented in a 12-min, easy-to-follow audio file and a follow-along transcript.	
5. **Normalization/experiential** context using the *“*Cancer Survival Toolbox: An Audio Resource Program*”* In this National Cancer Institute golden star award program, patients with specific cancer concerns demonstrate practical treatment tools for daily life.	
6. **Values clarification throughout the treatment decisions** through the use of a balance sheet, a simple table identifying the pros and cons for self and others (family and friends), resulting in values clarification for the patient-decision partner dyad. The balance sheet will be used during interactions within patient-decision partner dyads and with the CPN and oncology providers.	
7. **Structured time/data sharing** (i.e., balance sheet and HRQL-PSY graphical summaries) **with oncology providers** to support the oncology provider’s interactions with the patient-decision partner dyad and facilitate decisions.	

The decision-making tutorial is initially presented with a practice decision balance sheet (“telling others about cancer”) as the first decision and, in brief, as a “booster” for subsequent decisions [40]. The practice decision is meant to be less consequential and threatening than the three single-event decisions, allowing participants to practice decision skills. Once patients receive initial treatment options from their providers, they undertake values clarification using the balance sheets with the CPN before the next clinic visit. Structured time with the CPN and enhanced communication with oncology providers related to the decision process is an important intervention component, promoting multidisciplinary collaboration to address issues related to disease progression. Through this interactive process, the mHealth decision aid emphasizes informed, shared decision-making for patients, decision partners, the CPN, and oncologist through values clarification and decision preference. Reports of intervention adherence are collected from participants and the research team. The mHealth decision aid delivery approach allows patients to make informed, quality decisions by cancer care educational audio files, by immediate and over time HRQL-PSY graphical summaries, and by decision balance sheets, while including a support structure with the decision partner, CPN, and oncologist.

A participant has the option to withdraw from the study if they no longer wish to participate in the study, or if a patient dies during the study that is not related to the protocol, then he will be withdrawn from the study. Standard of care is allowed as part of the intervention, which includes pamphlets and brochures of the anti-cancer treatment that is provided by the healthcare provider, but oral, written, or recorded materials related to decision making are not permitted. There will be no blinding to group assignments with the exception of the data analysts. We learned from pilot studies that having CPNs dedicated to either the decision intervention group or enhanced usual care group alleviates treatment diffusion issues.

### Data collection and study measures

Data collection is implemented in a secure database system designed at the UVa and is accessible only to limited internet-provided addresses. All data are kept on an encrypted server and participants are given a unique identifier. The minimum necessary number of research team members is given access to the data to help protect participant confidentiality. The measures are collected using mobile devices, allowing the research team to be flexible about where the data are collected and to adapt to patient and decision partner preferences regarding data collection. The research team monitors for participant and decision partner distress signs (e.g., fatigue, anxiety) and, to monitor participant burden on an ongoing basis, probes for feedback about participant reactions. Although depression and other signs of psychological stress are not considered an adverse event, if a patient or decision partner is determined to be at risk for depression—defined as a score of 16 or greater on the Center for Epidemiologic Studies Depression Scale [45]—they are referred to a mental health specialist. A member of the research team meets with each dyad (EUC and DI) for approximately 1 h at baseline and 20 min at each 3-month visit over the 9 months during which participants are enrolled in the study. All participants receive regular in-person or telephone contact by the CPN. This serves the purpose of helping participants access needed resources and keeps them engaged in the study to help retention. The mHealth delivery approach allows patients to make informed, quality decisions via decisional balance sheets, an audio cancer decision-making tutorial, and HRQL-PSY graphical summaries.

### Outcome variables

The primary outcome measures are as follows: (1) decisional conflict (uncertainty subscale), (2) decision regret, (3) HRQL-PSY graphical summary results, and (4) decision-making participation preference. Table 3 provides the domain, measurement, metric, method of aggregation and timepoint of each outcome variable using a previously described framework [46, 47]. A battery of self-report measures is collected from the patient-decision partner dyad at baseline and post-intervention. DI participants are given the intervention and HRQL-PSY at baseline and at 3-month intervals over the 9-month period.

**Table 3:** Outcome measures

Outcome	Domain	Specific measurement	Specific metric	Method of aggregation	Timepoint
Decisional conflict	Decisional conflict	Decisional Conflict Scale, Uncertainty Subscale	Change from baseline	Mean	Baseline, 9 months
Decisional regret	Regret	Decision Regret Scale	Value at timepoint	Absolute number	9 months
Health-related quality of life	Health-related quality of life	Prostate Cancer Symptom Scale	Change from baseline	Mean	3, 6, and 9 months
Decision making participation	Decision making participation	Control Preference Scale	Change from baseline	Mean	3, 6, and 9 months

Fully specified outcomes based on previously described clinical trials outcome reporting framework [47, 48]

At the end of the approximately 9-month period, DI participants complete an mHealth decision aid evaluation to capture perceptions related to communications and overall decision satisfaction. The qualitative portion of the study includes a subset of the DI dyads (*n* = 30), 15 of which are expected to enrich data collection and help capture patients’ and decision partners’ perspectives regarding intervention delivery and the role of the CPN. The topics that are explored during these post-intervention semi-structured interviews include the following: (1) decisional changes over time between the patient and decision partner, (2) intervention utility, (3) patient-decision partner dyad and oncologist interactions, and (5) future implementation recommendations.

### Statistical analysis and power calculation

The main goal of this mixed-methods research is to test whether the decision support intervention is performing as hypothesized and warrants further research; thus, one-sided testing will be employed. The analysis plan assumes accrual (*N* = 158 dyads) for the quantitative portion of the study and a 15% dropout rate and assumes that the research team will have complete data on 134 dyads, providing approximately 89% power to detect an effect size of 0.5 for change in the main outcome measures for the DI group compared to no change in the EUC group using case-wise deletion. These calculations are informed by pilot data. Considering not all participants will be a dyad, and a smaller number will be single participants, it is unlikely that this would affect the power of the study. This will be reported in future dissemination materials once the study is completed.

For quantitative measures, descriptive analyses will be conducted as appropriate. Change in primary outcomes before and after the intervention will be assessed with analysis of covariance models that include the stratification factors (race and decision event), time, intervention arm, and a time-by-intervention-arm interaction. Additional analyses will assess whether the magnitude of change in primary outcome variables is similar between each member of the dyad using regression models that account for the correlation between each dyad and within participants over time. *Intention-to-treat* is defined as the analysis of all eligible patient-decision partner dyads by treatment as randomized, regardless of compliance with the intervention. Depending on the extent to which the data contain missing data, imputation will be considered for both the dyads and individual participants. Two methods will be used: (1) primary results will be based on an intention-to-treat analysis so that all eligible patients who entered the study will be included in the analyses regardless of compliance and (2) analyses will also include analysis of evaluable cases with more than 80% compliance (operationalized as completed all visits, all parts of the intervention but the home module, and had no other protocol violations) [48]. Using intention-to-treat analysis takes into consideration the bias of dropouts, but using both analyses provide more confidence in the conclusions of the trial if both analyses produce similar effects [48].

Qualitative data will be transcribed, coded into strips, and categorized by observed similarities [49]. The relationships of the categories will then be examined to construct themes, and the representativeness of these themes will be reviewed by the research team. Procedures will be employed to enhance validity, including modified member checking, dual coding, and peer/expert review.

Data quality will be audited regularly by the research team. No interim analyses will be performed. Any change to the protocol will be agreed upon between the investigators and filed with their respective IRB. This study has been deemed of minimal risk; therefore, a Data and Safety Monitoring Board has not been convened. Site visits will be conducted as necessary to ensure data and implementation quality. Adverse event reporting and monitoring is handled by the Principal Investigator and Institutional Review Board at each study site, and reviews will occur at least annually. The PI will report the event the IRB and Cancer Center Trials Office within 5 business days. All harms or adverse events (e.g., death unrelated to study intervention) are collected systematically and will be reported in trial publications, with consideration of maintaining patient privacy and confidentiality. Due to the unlikely event of harm related to this intervention, there are no plans to compensate participants for medical expenses, lost wages, disability, or discomfort. The charges for any medical treatment they receive will be billed to their insurance. The participants will be responsible for any amount of their insurance that is not covered.

No publication restrictions exist for this trial. All suggested topics for presentation or publication related to the trial will be sent to the PI of the study. There will be a discussion between PI and research team about the topic(s) for dissemination and where the dissemination items will be sent for review and potential acceptance. A query of those who are interested in substantially contributing to the presentation or publication will be made by the designated lead author. Assignments and contributions for the presentation or publication are made with the consideration of the authors’ expertise. There is no plan to utilize professional writers in the development of presentations or publications from this protocol.

## Discussion

Despite advances in the available treatment options for patients with advanced prostate cancer, supportive interventions directed towards complex treatment-based decisions made by patients and their decision partners have not been explored in depth. Decision aids have shown promise in oncological disease states [21], but their use has been limited in patients with advanced cancer. Decision partners are a valuable resource to be considered when attempting to assist patients through the process of cancer treatment decision-making. A key strength of the CHAMPION intervention is that decision partners are direct recipients of the intervention. Interventions to engage patients and decision partners represent a novel, sustainable, and scalable way to build on individual and supporter strengths.

The innovative nature of the proposed decision aid is enhanced by mHealth technology and intervention delivery by trained CPN. Low decision aid use, despite well-demonstrated utility, may be a result of dependence on healthcare professional administration. This is likely to be an ongoing barrier, particularly given the increasing demands on provider time and availability. The CHAMPION intervention CPNs are non-healthcare professionals from the patient and decision partner community who receive training to assist patients with various aspects of promoting and maintaining health, as well as in the conduct of research [30, 31, 37]. The National Academy of Medicine [50] (formerly the Institute of Medicine) recommends expanding CPN use and evaluation to improve care access and quality of care, especially among medically underserved and racial/ethnic minority communities.

Minorities with advanced cancer, particularly African Americans, are often faced with ineffective health-related communication in regard to receiving adequate end-of-life care, which may result in overly aggressive, unwanted care [51–53]. Thus, there is a need for a feasible and interactive intervention to improve communication between minorities and healthcare professionals [26, 27]. The CHAMPION intervention incorporates within the balance sheet narratives a multi-faceted approach. This approach utilizes the patient’s decision partner, CPN, and oncology providers for support and decision-making empowerment. This approach also reinforces the important values identified by minority patients and their decision partners, such as spirituality, family, and trusted others [20]. The ability for patient-decision partner dyads to identify and discuss their own priorities using the decision balance sheets—combined with HRQL-PSY graphical summaries and listening to the cancer decision-making audio tutorial—provides a comprehensive decision-making intervention for both the patient and their decision partner. The integration of these values in the mHealth decision aid intervention creates a tailored intervention for each pair, particularly for those from minority groups. The intervention may support minorities in expressing important concerns with their oncology providers, thereby facilitating a clearer treatment decision-making process and improving outcomes.

This novel and feasible intervention approach, incorporating a CPN to administer an mHealth decision aid to patients with advanced prostate cancer and to their decision partners, should assist oncology providers, policymakers, and the public in sustainably improving healthcare system structures to support complex treatment decision-making. CHAMPION procedures and outcomes have the potential to promote better HRQOL, reduce decisional conflict and decision regret in persons affected by advanced prostate cancers in a community and family-centered care context, and, ultimately, to contribute to reducing the disparities in cancer healthcare treatment.

## Trial status

Protocol date and version: August 24, 2018, version 1.11

Date recruitment begin: June 2, 2017

Approximate date of recruitment completion: September 1, 2021

## Supplementary Information

**Additional file 1.** SPIRIT Checklist

**Additional file 2.** World Health Organization Trial Registration Data Set

## Data Availability

Upon reasonable request of the corresponding author, qualified investigators will have access to the dataset under a Data Use Agreement.

## Abbreviations

CHAMPION: Cancer Health Aid to Manage Preferences and Improve Outcomes through Navigation
CPN: Community patient navigator
DI: Decision Intervention
EUC: Enhanced usual care
HRQOL-PSY: Psychosocial health-related quality of life
IRB: Institutional Review Board
JHU: Johns Hopkins University
mHealth: Mobile health
QoL: Quality of Life
UVa: University of Virginia
VCU: Virginia Commonwealth University
