# Supplementary material for: Employing a mobile health decision aid to improve decision-making for patients with advanced prostate cancer and their decision partners/proxies: the CHAMPION randomized controlled trial study design
Source: Trials. 2021 Sep 16;22:631. doi: 10.1186/s13063-021-05602-0 (PMC8444368; doi:10.1186/s13063-021-05602-0)
Supplement: Supplementary file 2 — Additional file 2. World Health Organization Trial Registration Data Set [file 13063_2021_5602_MOESM2_ESM.docx]

**Table 3. World Health Organization Trial Registration Data Set (Version 1.3.1)**

|  | Item | Information |
| --- | --- | --- |
| 1 | Primary Registry and Trial Identifying Number | National Clinical Trials Registry NCT03327103 |
| 2 | Date of Registration in Primary Registry | 10/31/2017 |
| 3 | Secondary Identifying Numbers | National Institutes of Health National Institute of Nursing Research, R016483-01A1 |
| 4 | Source(s) of Monetary or Material Support | National Institutes of Health |
| 5 | Primary Sponsor | University of Virginia, Dr. Randy Jones |
| 6 | Secondary Sponsor(s) | Johns Hopkins University, Dr. Jennifer Wenzel |
| 7 | Contact for Public Queries | Professor Randy A. Jones, PhD, RN, FAAN  University of Virginia School of Nursing  202 Jeanette Lancaster Way  PO Box 800782  Charlottesville, VA 22908  +1 (434) 924-0125  rajc@virginia.edu |
| 8 | Contact for Scientific Queries |  |
| 9 | Public Title | A Decision Aid for Advanced Prostate Cancer Patients and their Decision Partners |
| 10 | Scientific Title | Employing a Mobile Health Decision Aid to Improve Decision Making for Patients with Advanced Prostate Cancer and their Decision Partners/Proxies: the CHAMPION Randomized Controlled Trial Study Design |
| 11 | Countries of Recruitment | United States of America |
| 12 | Health Condition Studied | Advanced Prostate Cancer |
| 13 | Interventions | An interactive mHealth decision aid that includes immediate and over time quality of life (QL) graphical summaries for advanced prostate cancer patients to enhance informed, shared decision making and will also be used to assess the CPNs' role in the decision-making process. |
| 14 | Inclusion and Exclusion Criteria | Inclusion:  a diagnosis of stage III/IV prostate cancer, life expectancy of ≥ 6 months, Karnofsky Performance Status [45] ≥60, age ≥18 years, and the ability to understand English.  Exclusion  severe psychiatric problems, prior non-prostate malignancy in the past 3 years (except treated basal cell/squamous cell skin cancer or superficial bladder cancer). |
| 15 | Study Type | Randomized open label parallel assignment supportive care intervention study |
| 16 | Date of first enrollment | July 7, 2017 |
| 17 | Sample Size | 134 dyads (268 participants) |
| 18 | Recruitment status | Recruiting |
| 19 | Primary outcomes | Decisional Conflict  Decision control preference  Decision regret  Health-related Quality of Life |
| 20 | Secondary outcomes | Semi-structured interviews 9 months after enrollment  Qualitative evaluation of community patient navigator role in delivery of decision aid |
| 21 | Ethics Review | Approved |
| 22 | Completion date | NA |
| 23 | Summary results | NA |
| 24 | Individual clinical trial participant-level data sharing statement | Sharing Statement: Final research data will be shared openly and timely in accordance with current National Institutes of Health guidelines (http://grants.nih.gov/grants/policy/data_sharing/) with consideration for protection of the participants confidentiality and privacy at all times. |
|  | | |
